# Device-measured postpartum sleep and breastfeeding contexts among women in urban Vietnam

**DOI:** 10.3389/fpubh.2026.1887659

**Published:** 2026-08-25

**Authors:** Yunhee Kang, Minsol Kim, Toan Thi Nguyen, Ahreum Choi, Heeyeon Kim, Tuan Minh Vo

**Affiliations:** 1 Department of Food and Nutrition, Seoul National University, Seoul, Republic of Korea; 2 Department of International Health, Johns Hopkins School of Public Health, Baltimore, MD, United States; 3 Yonsei Institute for Global Health, Yonsei University Health System, Seoul, Republic of Korea; 4 OB-GYN Department, University of Medicine and Pharmacy at Ho Chi Minh City, Ho Chi Minh City, Vietnam; 5 Graduate School of Public Health, Seoul National University, Seoul, Republic of Korea; 6 Graduate School of Public Health and Healthcare Management, The Catholic University of Korea, Seoul, Republic of Korea

**Keywords:** breastfeeding, postpartum, postpartum sleep, sleep environment, Vietnam

## Abstract

**Introduction:**

Postpartum sleep patterns are embedded within complex caregiving environments, including infant feeding, nighttime caregiving demands, and maternal recovery. This cross-sectional study aimed to characterize maternal sleep patterns and explore variation in sleep across infant feeding contexts, exclusive and partial breastfeeding practices among women at 1 month postpartum in Ho Chi Minh City, Vietnam.

**Methods:**

Sleep patterns were assessed using a commercial wearable device, and reported breastfeeding indicators included exclusive breastfeeding (EBF) status, feeding frequency during day and nighttime, and breast milk expression practices. Adjusted linear and logistic regression analyses were used, adjusting for covariates.

**Results:**

The average daily sleep duration was 5.53 hours (SD: 1.36), falling substantially below adult recommendations. The prevalence of EBF was 47.7%, with mothers breastfeeding an average of 6.8 times (SD: 1.3) during the day and 2.8 times (SD: 0.6) at night. After adjusting for socio-demographic, reproductive, and caregiving-related covariates, feeding group was not significantly associated with either device-measured sleep duration (adjusted β = −0.31, 95% CI: −0.68, 0.06) or the likelihood of sleeping ≥5 hours (adjusted PR = 0.94, 95% CI: 0.65, 1.36). Also, there were no statistically significant associations between daytime or nighttime breastfeeding frequency or pumping frequency and sleep duration.

**Discussion:**

These findings highlight the complexity of postpartum sleep within real-world caregiving environments, suggesting that factors beyond feeding practices alone may contribute to variation in maternal sleep. This study provides empirical insights into maternal sleep in an urban LMIC setting and highlights the growing role of wearable devices in assessing postpartum sleep.

## Introduction

Maternal sleep has been identified as an important factor influencing postpartum recovery, maternal wellbeing and caregiving behaviors. Maternal sleep occurs within complex caregiving ecologies shaped by the interrelated influences of breastfeeding practices, nighttime caregiving demands, family support, room-sharing arrangements, maternity leave, labor conditions, and exposure to commercial formula-feeding environments (1–3).

Previous studies suggest a complex, potentially bidirectional relationship between maternal sleep patterns and breastfeeding practices. Through hormonal responses that promote relaxation and sleep quality, the release of oxytocin and prolactin during breastfeeding may lead to feelings of calmness and drowsiness (4). A meta-analysis reported that breastfeeding mothers have a 0.24 h longer duration of sleep than non-breastfeeding mothers (5). Maternal sleep quality and duration may play a critical role in breastfeeding behaviors. Poor sleep has been linked to physical exhaustion, reduced maternal self-efficacy, and an increased likelihood of early cessation of breastfeeding (6). Poor maternal sleep may also compromise the frequency of breastfeeding sessions, potentially influencing milk supply and infant nutrition (7), and has been associated with various adverse health outcomes, such as postpartum depression (8) and accelerated biological aging (9). While these findings highlight the interconnectedness of sleep and breastfeeding, most existing studies have been conducted in high-income countries. Evidence examining these associations in low- and middle-income countries remains limited, particularly in Southeast Asia.

In Vietnam, rapid urbanization, women's high employment, and multigenerational caregiving norms highlight complex childcare environments (10). Although women are legally entitled to 6 months of maternity leave (11), many women did not take leave, and utilization of maternity leave varies substantially, particularly in urban settings. Many women remain engaged in income-generating activities during the early postpartum period, which may constrain opportunities for rest and recovery. In Vietnam, exclusive breastfeeding (EBF) rate (0–5 months) rose from 24 % in 2014 to 45 % in 2020 (12). In a study in Ho Chi Minh City, only 32% of study mothers practiced the recommended 6 months of EBF (13). Although breastfeeding is traditionally valued in Vietnam, the continuation of EBF for the recommended 6 months is uncommon (14). National surveys indicate that while initiation rates are relatively high, many mothers introduce complementary foods or breastmilk substitutes earlier than recommended (15). These structural and social factors may shape both infant feeding practices and maternal sleep patterns. Although the Vietnamese government has adopted the Decrees based on the International Code of Marketing of Breast-milk Substitutes (16), commercial baby formula is widely used in urban Vietnam (17).

This study aims to examine the association between maternal sleep duration and breastfeeding practices within the context of postpartum caregiving demands in rapidly urbanizing Ho Chi Minh City, Vietnam. Specifically, we have two aims: 1) to compare device-measured maternal sleep duration across breastfeeding behaviors; and 2) to examine how breastfeeding behaviors (feeding frequency and milk expression) are associated with maternal sleep patterns.

To improve the accuracy of sleep duration, this study introduced commercial wearable devices. The integration of wearable activity trackers allows for passive, non-invasive measurement of physical activity and sleep (18). Wearable activity trackers, particularly actigraphy, have been used to assess maternal and infant sleep for more than a decade (18). However, the use of consumer-grade devices such as Fitbit, which were originally designed for personal wellness rather than scientific research, has only recently been applied in women's sleep studies during pregnancy and lactation (19). To our knowledge, this study is one of the first studies in Vietnam to utilize commercial wearable devices in assessing sleep patterns among postpartum women. Findings from this study may inform future maternal health and breastfeeding support programs in similar urban LMIC settings.

## Methods

### Study design and participants

This Empowering Mothers and Improving Breastfeeding (EMIB) study was designed as a cross-sectional study to investigate the association between device-measured sleep patterns and breastfeeding practices among postpartum mothers in Ho Chi Minh City, Vietnam (20). A total of 369 postpartum mothers, aged 19–49 years, with infants aged 1 to < 2 months, were initially recruited from the Obstetrics and Gynecology Department and the Healthy Child Clinic at University Medical Center (UMC) Hospital, Ho Chi Minh City (UMP), one of the largest tertiary referral hospitals in southern Vietnam.

**Figure 1:**
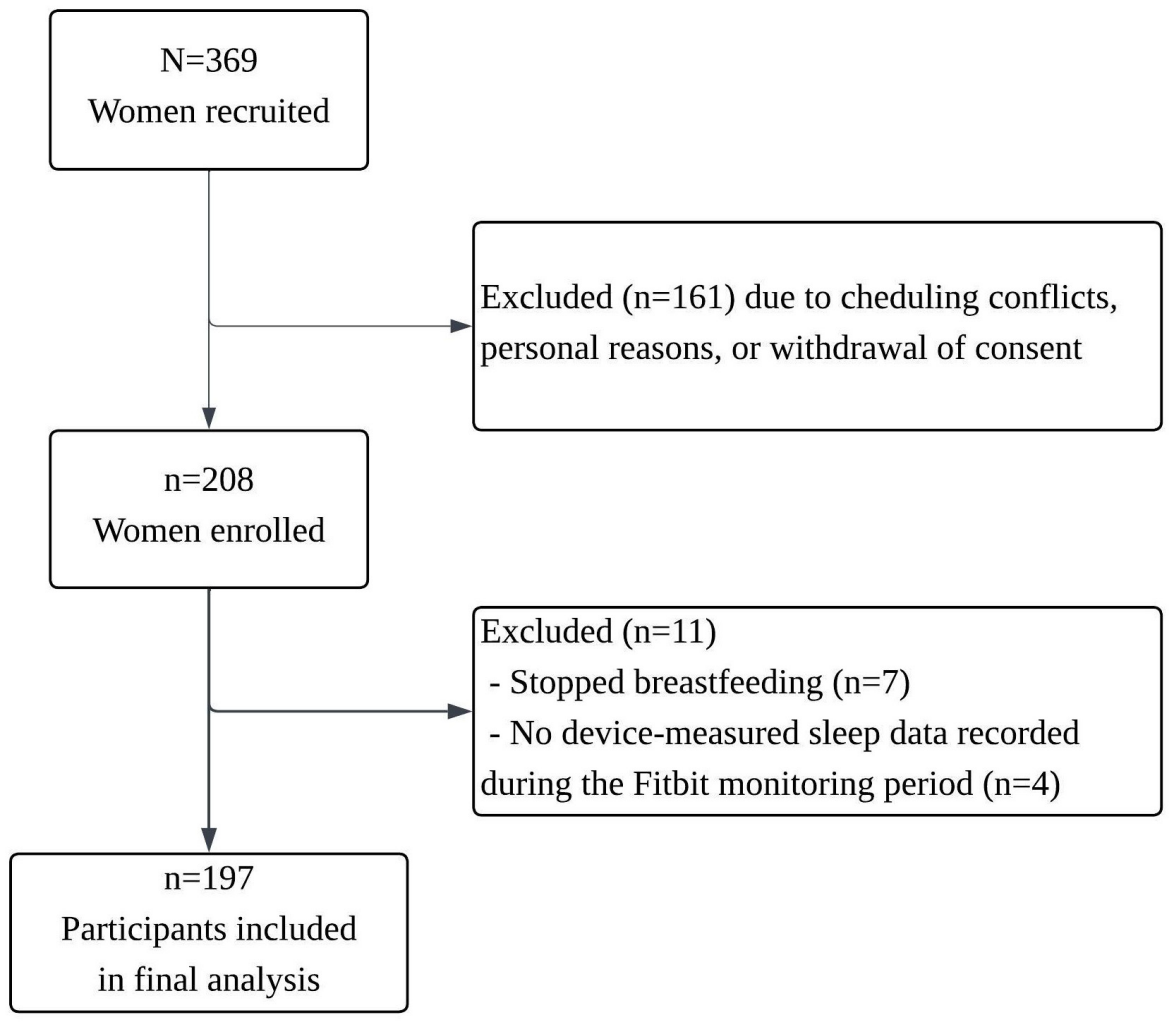
Flow diagram.

Of the 369 mothers who expressed initial interest, 161 did not proceed at the household visit stage due to scheduling conflicts, personal reasons, or withdrawal of consent (Figure 1). The remaining 208 mothers completed enrollment, received wearable devices, and were included in the final study sample.

Two UMP research coordinators conducted the recruitment process with mothers who came to the UMC hospital for routine check-ups. The coordinators provided information to women with a leaflet outlining the study objectives and eligibility. Those mothers who were interested in the study were invited to a private consultation room for the consent process. The research coordinators read the consent form, including study objectives, detailed data collection process, benefits and harm, and volunteer participation, and obtained written informed consent.

### Sample size

The original sample size was calculated based on the prevalence of EBF among mothers in urban Vietnam. The prevalence of EBF in urban areas was estimated at about 86% at 1 month (21). Using a single-proportion formula for cross-sectional studies with a 95% confidence level and 5% margin of error, the required sample size was 196 participants. To account for potential non-response or incomplete data, the target sample size was expanded, and 208 mothers were included in the analysis. With the final analytic sample (*n* = 197; 94 exclusively breastfeeding and 103 partial breastfeeding mothers), and assuming a standard deviation of 1.25 h, the study had approximately 80% power (two-sided α = 0.05) to detect a between-group difference of 0.5 h in total sleep duration, the primary outcome Analyses examining other breastfeeding behaviors and binary sleep outcomes were considered exploratory because the study was not specifically powered for these analyses.

### Questionnaire

The questionnaire covered a range of topics, including sociodemographic characteristics (i.e., women's age, husband age, household size, marital status, occupation, highest education attained, household income, religion, ethnicity), pregnancy and delivery history (i.e., parity, delivery mode), use of maternity leave, breastfeeding practices, sleep behaviors and environment, maternal diet, physical activity, and maternal mental health assessed through the Edinburgh Postnatal Depression Scale (EPDS) (22). The questionnaire was prepared in English and translated into Vietnamese. The form was prepared as ODK form in KOBO Tool (https://www.kobotoolbox.org/).

### Application of a commercial wearable device

The Fitbit Charge 6 Premium Fitness Tracker, equipped with a tri-axial accelerometer and optical heart rate monitor, was used to assess participants' sleep patterns and physical activity. Fitbit Charge 6 (Fitbit Inc., San Francisco, CA, USA) devices contained microelectromechanical tri-axial accelerometers and proprietary algorithms to provide accurate step counts (23). The Fitbit devices provided detailed measurements, including sleep stages: Light, Deep, and Rapid Eye Movement (REM) sleep, sleep scores, and stress management indicators, with a battery life of up to 7 days. Several validation studies have demonstrated acceptable accuracy and sensitivity of Fitbit-derived sleep estimates compared to polysomnography (24). In a recent study on a similar topic, Fitbit Charge 5 demonstrated moderate validity in measuring overall sleep duration and offered clinically acceptable estimates for long-term monitoring, despite some limitations in detecting brief awakenings and specific sleep stages (25).

Initially, research coordinators visited study participants' homes to deliver the wearable devices, assist with setup, and guide participants in downloading the Fitbit application. To improve participant retention, the protocol was modified so that eligible mothers were fitted with the Fitbit device immediately after providing informed consent at the hospital.

Before distribution, individual Google accounts were created in advance and linked to specific Fitbit devices. On the day of allocation, coordinators connected each device to its corresponding Google account. Each participant's Fitbit device was synchronized with each study participant's smartphone through the Fitbit mobile application. Participants were instructed to wear the device continuously throughout three consecutive days and to remove it only when necessary, such as during showering. Most participants appeared to comply with the study protocol. While occasional nighttime device removal may have occurred, these instances were not systematically documented.

On the fifth day, research coordinators returned to collect the device, administer a survey, and perform anthropometric measurements of both mother and child during home visits. Each visit lasted approximately 40 min. Sleep data were synchronized with the Fitbit mobile application and extracted as daily summary measures. On the scheduled data collection day, trained research staff accessed each participant's Fitbit account through the Fitbit mobile application and recorded sleep-related metrics, including total sleep duration, sleep onset latency, and other available sleep parameters. Device-measured total sleep duration was defined as the total amount of sleep accumulated within a 24-h period, including both nighttime sleep and daytime naps automatically detected by the device.

### Data collection

A pilot study was conducted from June 25 to July 1, 2024, to assess the feasibility of the study protocol and identify logistical and technical challenges prior to the main data collection. The main data collection took place from July 29 and continued to December 18, 2024.

### Dependent variable: maternal sleep patterns

Maternal sleep patterns were assessed using two approaches: device-measured data from Fitbit Charge 6 and recall-based self-reports. The Fitbit device records sleep when at least 1 h of sleep duration is detected and classifies sleep stages into light, deep, REM, and awake. Light sleep duration is the time spent in N1 and N2 stages of sleep, where heart rate slows and the body begins to relax. Deep sleep duration is the time spent in stage N3 (slow-wave sleep), characterized by minimal responsiveness to external stimuli and delta wave activity. REM sleep duration is the time spent in rapid eye movement sleep, during which brain activity increases and vivid dreaming commonly occurs. Total sleep duration was calculated as the cumulative duration of light, deep, and REM sleep within a 24-h cycle. Mothers provided recall-based reports of their sleep: average nighttime sleep durations (in hours from 8 pm to 5 am), the time it typically took to fall asleep (in minutes), daytime napping behavior (presence and duration from 5 am to 8:00 pm), and experience of repeated nighttime awakenings or sleep disturbances.

### Independent variables: breastfeeding practices

Breastfeeding behaviors were assessed by an interviewer-administered questionnaire. Mothers were asked to report the frequency of breastfeeding during the daytime and nighttime on the previous days, as well as the average duration of a typical breastfeeding session. Exclusive breastfeeding is defined as whether the baby was fed only breast milk, without any other foods or liquids. Partial breastfeeding is defined as providing breast milk alongside formula feeding. Frequency of breastfeeding is defined as the number of breastfeeding sessions during the day and night. Breastfeeding duration per session indicates the average time spent breastfeeding. Breastfeeding continuation is defined as whether the mother has completely stopped breastfeeding and pumping milk. Pumping practices included frequency of pumping, volume of milk expressed, and regularity of pumping schedules.

**Sleeping-related behaviors and caregiving arrangements** included daytime nap, repeated waking up, and sleeping with baby and other family members. We assessed the duration of daily caregiving and household labor.

### Statistical analysis

Out of 208 enrolled mothers, 197 women who were breastfeeding at the time of the survey and had at least one device-measured sleep record were included in the analytic sample. Eleven women were excluded as they stopped breastfeeding (*n* = 7) or had no device-measured sleep data recorded during the Fitbit monitoring period (*n* = 4) (Figure 1). A total of 772 device-measured sleep records were available from 197 mothers. The first day of wearing (*n* = 86 observations) as Fitbit records sleep only from midnight on the first day. The last wear day, either day 5 (*n* = 127 observations) or day 6 (*n* = 16 observations), was also excluded since daytime nap data may be incomplete and home visits by coordinators for device retrieval may influence participants' daily routines. As a result, valid total sleep duration data were available for 180 participants on day 2, 176 on day 3, 166 on day 4, and 14 on day 5. Mean sleep duration was 5.45 h (SD: 1.85) on day 2, 5.68 h (SD: 1.69) on day 3, 5.58 h (SD: 1.93) on day 4, and 5.40 h (SD: 1.95) on day 5. Individual-level mean sleep duration was calculated by averaging all available valid measurement days between the first and final wear days. Overall, 9 mothers had one valid day of sleep measurement, 44 had two valid days, 137 had three valid days, and 7 had four valid days.

An exploratory analysis was conducted to present mean (SD) for continuous variables and *n* (%) for categorical variables for independent and dependent variables and all covariates. Differences in participant characteristics, breastfeeding practices, and sleep-related behaviors between exclusive and partial breastfeeding groups were evaluated using Student's *t*-tests for continuous variables and chi-square tests (or Fisher's exact tests when expected cell counts were less than five) for categorical variables. Linear regression analysis was used to test the association between breastfeeding behaviors (exclusive vs. partial breastfeeding, daytime or nighttime breastfeeding frequency, ever pumping breastmilk, and pumping frequency) and device-measured total sleep duration. Device-measured sleep duration was categorized as a binary variable (≥ 5 h or < 5 h), and associations were assessed using modified Poisson regression models to estimate prevalence ratios (PR), based on a sample mean of 5.5 h among study participants. Although 6 h is commonly used as a cut-off for defining short sleep in the general adult population (26), we applied a 5–h threshold in this study to reflect more severe sleep restriction, typical of the early postpartum month, which often averages only 5–6 h of total sleep (27).

Adjusted regression accounted for women's age, use of maternity leave, delivery mode (normal, c-section), household income, highest education attained, number of children under 5 years old, and ethnicity. Covariates were selected based on prior literature and conceptual relevance to maternal sleep and breastfeeding behaviors. Additional sensitivity analyses were conducted to evaluate the robustness of findings. Modified Poisson regression models were applied for alternative short-sleep thresholds of ≥6 or ≥7 h. Linear mixed-effect models using all available valid sleep records were fitted to account for repeated measurements and unequal numbers of valid measurement days.

### Ethical considerations

The study protocol was approved by the Board of Ethics in Biomedical Research at the University of Medicine and Pharmacy in Ho Chi Minh City (Approval No. 619/UMP-BOARD) on May 2, 2024. Written informed consent was obtained from all participants before data collection.

## Results

Out of 197 women, 94 (47.7%) mothers exclusively breastfed, and 103 (52.3%) were partially breastfeeding. The mean age of mothers was 30.4 years (SD: 3.9), while the mean age of their husbands was 32.8 years (SD: 5.5). The average household size was 4.9 members (SD: 1.5) (Table 1). Most participants (96.5%) had completed college/university/postgraduate degrees, and almost all (99.0%) were married. For occupation, 42.7% were employed in the private sector, 18.7% were government employees, 37.4% were self-employed, and 1.2% were entrepreneurs or engaged in other types of work. 28.9% reported an income of less than 49,999,999 VND, while 22.8% earned more than 70,000,000 VND. Regarding religion, 92.9% of participants reported having no religion. For ethnicity, 93.9% of participants were Kinh, while 6.1% were Hoa or members of other minority groups.

**Table 1:** Socio-economic characteristics among exclusive breastfeeding vs. partial breastfeeding mothers (*n* = 197).

Characteristics	Total (*n* = 197)	Exclusive breastfeeding (*n* = 94)	Partial breastfeeding (*n* = 103)	*p*-val^1^
Mother Age, mean (SD)	30.4 (3.9)	30.2 (4.1)	30.5 (3.7)	0.63
Husband Age, mean (SD)	32.8 (5.5)	32.9 (5.9)	32.7 (5.1)	0.78
HH size, mean (SD)	4.9 (1.5)	4.8 (1.4)	4.9 (1.5)	0.87
Education, *n* (%)				
College/University/Postgraduate	190 (96.5)	94 (100.0)	96 (93.2)	0.01
Upper or higher than upper secondary	7 (3.5)	0 (0.0)	7 (6.8)	
Marital status, *n* (%)				
Married	195 (99.0)	93 (98.9)	102 (99.0)	0.37
Divorced/single	2 (1.00)	1 (1.1)	1 (1.0)	
Occupation, *n* (%)				
Employee (Government)	32 (18.7)	15 (17.6)	17 (19.8)	0.14
Employee (Private Sector)	73 (42.7)	30 (35.3)	43 (50.0)	
Entrepreneur/Other	2 (1.2)	1 (1.2)	1 (1.2)	
Self-Employed (Agriculture/Fishing)	64 (37.4)	39 (45.9)	25 (29.1)	
Income, *n* (%)				
< 49,999,999 VND	57 (28.9)	23 (24.5)	34 (33.0)	0.12
50,000,000 to 59,999,999 VND	55 (27.9)	22 (23.4)	33 (32.0)	
60,000,000 to 69,999,999	40 (20.3)	23 (24.5)	17 (18.5)	
>70,000,000 VND	45 (22.8)	26 (27.7)	19 (18.5)	
Religion, *n* (%)				
Buddhist	10 (5.1)	2 (2.1)	8 (7.8)	0.11
Catholic	4 (2.0)	3 (3.2)	1 (1.0)	
No religion	183 (92.9)	89 (94.7)	94 (91.3)	
Ethnicity, *n* (%)				
Kinh	185 (93.9)	92 (97.9)	93 (90.3)	0.02
Hoa/another minority	12 (6.1)	2 (2.1)	10 (9.7)	
Pregnancy and delivery history				
Ever miscarriage, *n* (%)	29 (13.9)	10 (10.6)	17 (16.5)	0.23
Number of deliveries, *n* (%)				
One time	118 (59.9)	54 (57.5)	64 (62.1)	0.41
Twice	72 (36.5)	35 (37.2)	37 (35.9)	
Three times	7 (3.5)	5 (5.3)	2 (1.9)	
Age of the first pregnancy, mean (SD)	28.4 (3.6)	28.2 (3.3)	28.6 (3.9)	0.48
Gestational age (weeks) at pregnancy outcome, mean (SD)	38.1 (1.4)	38.1 (1.5)	38.2 (1.3)	0.62
Diabetes pregnancy, *n* (%)	33 (16.8)	15 (16.0)	18 (17.5)	0.78
Delivery mode, *n* (%)				
Normal delivery	94 (47.7)	43 (45.7)	51 (49.5)	0.60
C-section (planned or unplanned)	103 (52.3)	51 (54.3)	52 (50.5)	
Maternity leave taken, *n* (%)				
No	118 (59.9)	64 (68.1)	54 (52.4)	0.03
Yes	79 (40.1)	30 (31.9)	49 (47.6)	
Depressive symptoms based on EPDS, *n* (%)				
Depression not likely	192 (97.5)	92 (97.9)	100 (97.1)	0.73
Any depression	5 (2.5)	2 (2.1)	3 (2.9)	

EPDS, Edinburgh Postnatal Depression Scale. ^1^Tested by Student t-test for continuous variables and Chi2-test for categorical variables between exclusive breastfeeding and partial breastfeeding group.

A history of miscarriage was reported by 13.9% of participants. Regarding delivery history, 59.9% of women were first-time mothers, 36.5% had delivered two children, and 3.5% had three children. The mean age at first pregnancy was 28.4 years (SD: 3.6). The mean gestational age at delivery was 38.1 weeks (SD: 1.4), and 52.3% of mothers underwent cesarean sections (planned or unplanned). For maternity leave, 40.1% had paid or unpaid leave. Among mothers who did not take leave, 18.6% had no occupation generating income, while 80.4% had an income-generating occupation. Most characteristics are similar between exclusive breastfeeding mothers and partial breastfeeding mothers. Exclusive breastfeeding mothers had a higher education attainment (100.0% vs. 93.2%), belonged to the Kinh ethnic group (97.9% vs. 90.3%), and were less likely to take maternity leave (31.9% vs. 47.6%) than partially breastfeeding women (all *p* < 0.05).

### Breastfeeding practices

Exclusively breastfeeding mothers showed a higher frequency of daytime breastfeeding than partially breastfeeding mothers (7.2 times vs. 6.4 times; *p* < 0.001), while nighttime breastfeeding frequency did not differ (both groups: 2.8 times; *p* = 0.86) (Table 2). The mean duration of a single breastfeeding session was 28.0 min (SD: 6.5) among exclusively breastfeeding mothers and 25.9 min (SD: 8.0) among partially breastfeeding mothers (*p* = 0.04). A higher proportion of exclusively breastfeeding mothers reported ever having pumped breast milk compared with partially breastfeeding mothers (90.4% vs. 79.6%; *p* = 0.04). However, there was no significant difference in weekly pumping frequency between exclusive (6.4 times [SD: 8.9]) and partially breastfeeding mothers [5.9 times (SD: 8.9)] (*p* = 0.68).

**Table 2:** Recall-based breastfeeding practices and pumping patterns among exclusive and partial breastfeeding women (*n* = 197).

Characteristics	All (*n* = 197)	Exclusive breastfeeding (*n* = 94)	Partial breastfeeding (*n* = 103)	*p*-val^1^
Frequency of daytime breastfeeding, mean (SD)	6.8 (1.3)	7.2 (1.2)	6.4 (1.3)	< 0.001
Frequency of night-time breastfeeding, mean (SD)	2.8 (0.6)	2.8 (0.6)	2.8 (0.7)	0.86
Breastfeeding duration (min) at one session, mean (SD)	26.9 (7.4)	28.0 (6.5)	25.9 (8.0)	0.04
Ever pumping breastfeeding, *n* (%)	167 (84.8)	85 (90.4)	82 (79.6)	0.04
Frequency of weekly milk pumping, mean (SD) (*n* = 167)	6.1 (8.9)	6.4 (8.9)	5.9 (8.9)	0.68

^1^Tested by Student t-test for continuous variables and Chi2-test for categorical variables between exclusive breastfeeding and partial breastfeeding group.

### Device-measured sleep duration

The mean daily device-measured total sleep duration was 5.53 h (SD: 1.36), while the mean self-reported nighttime sleep time was 4.54 h (SD: 0.99; Median: 4 h) (Table 3). The proportions of mothers who slept ≥5, ≥6, and ≥7 h were 66.0%, 37.1%, and 11.2%, respectively.

**Table 3:** Device-measured and self-reported sleep patterns (*n* = 197).

*Device-measured sleep records*	Mean (SD) or *n* (%)
Sleep duration (hours)	5.53 h (1.36)
≥ 5 h sleep duration, *n* (%)	130 (66.0%)
≥ 6 h sleep duration, *n* (%)	73 (37.1%)
≥ 7 h sleep duration, *n* (%)	22 (11.2%)
Awake time	42.7 min (31.8)
Rapid eye movement (REM) sleep	69.3 min (30.5)
Deep sleep	53.5 min (25.1)
Light sleep	186 min (59.0)
*Self-reported sleep records*	
Sleep duration (hours)	4.54 h (0.99)

### Sleeping-related behaviors and caregiving arrangements

Some sleep-related behaviors were similar across feeding groups; 93.4% of mothers reported taking daytime naps, and 80.7% reported feeling sleepy or tired even after sleeping (Table 4). However, repeated nighttime awakenings were more common among mothers practicing exclusive breastfeeding compared to those practicing partial breastfeeding (96.8% vs. 84.5%, *p* = 0.003). Nearly all mothers slept with their baby (98.0%). Out of mothers who co-slept with their baby (*n* = 193), 9.3% slept with one additional family member and 90.2% slept with two or more family members (Table 4).

**Table 4:** Sleep-related behaviors and caregiving arrangements (*n* = 197).

Characteristics	All (*n* = 197)	Exclusive breastfeeding (*n* = 94)	Partial breastfeeding (*n* = 103)	*p*-val^1^
	*n* (%)	*n* (%)	*n* (%)	
Taking a nap	184 (93.4)	89 (94.7)	95 (92.2)	0.49
Feeling sleepy or tired even after getting sleep	159 (80.7)	79 (84.0)	80 (77.7)	0.26
Repeatedly waking up	178 (90.4)	91 (96.8)	87 (84.5)	0.003
Sleeping with a newborn in the same room	193 (98.0)	91 (96.8)	102 (99.0)	0.27
Sleeping with family members	*n* = 193	*n* = 91	*n* = 102	
Only with the baby	1 (0.5)	1 (1.1)	0 (0.0)	0.56
One family member	18 (9.3)	8 (8.8)	10 (9.8)	
Two or more family members	174 (90.2)	82 (90.1)	92 (90.2)	

^1^Tested by Student t-test for continuous variables and Chi2-test for categorical variables between exclusive breastfeeding and partial breastfeeding group.

The study women in both feeding groups reported substantial involvement in childcare activities; spending approximately 1–3 h per day on dressing, bathing, and feeding their children. Similar amounts of time were reported for playing with and carrying children. Yet less time was allocated to shopping (< 30 min per day) and to sitting and resting (30 min to 2 h) (Supplemental Table 1).

### Difference in sleep practices across feeding practices

**Figure 2:**
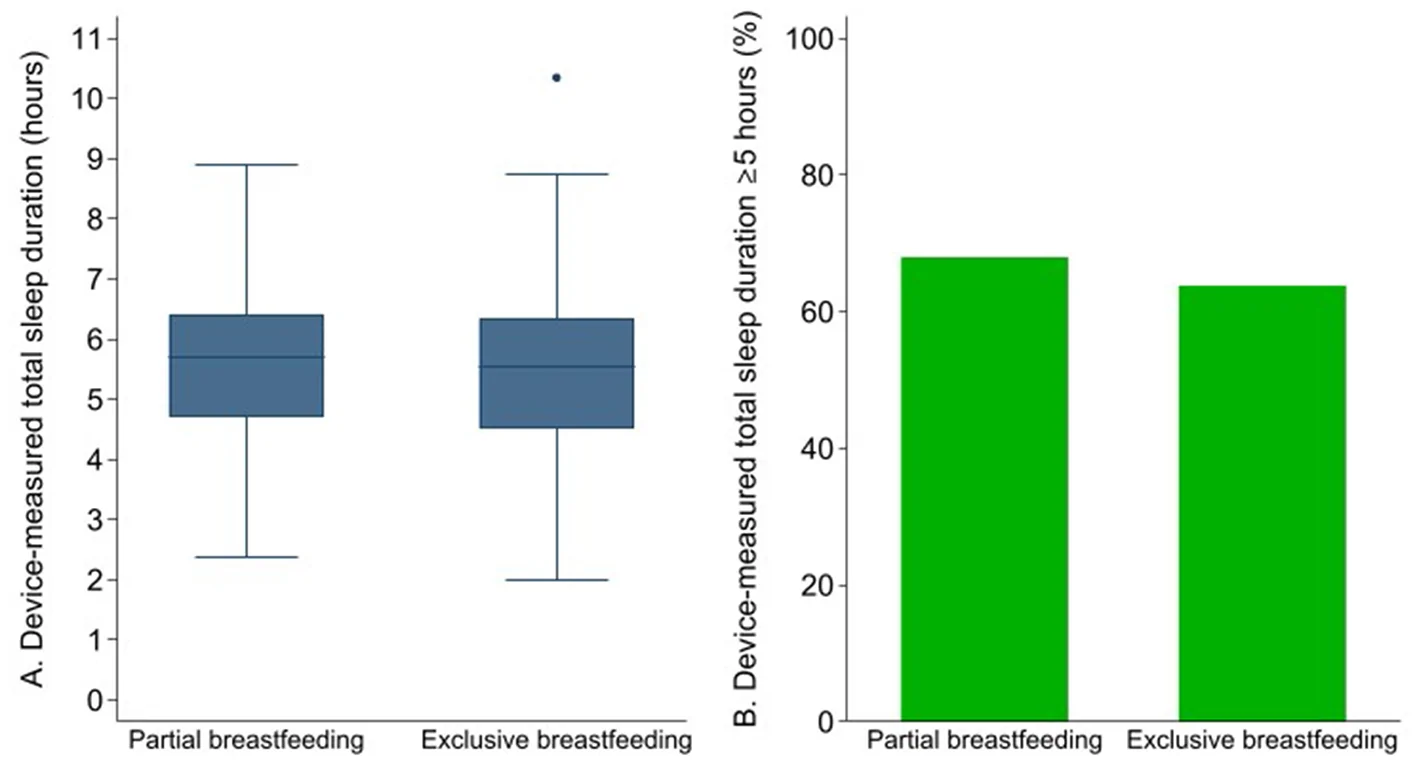
Comparison of device-measured sleep duration between exclusive and partial breastfeeding mothers: **(A)** *Mean total sleep duration (hours)*; **(B)** *Percentage of mothers with total sleep duration* ≥*5 h (%)*.

Exclusive breastfeeding mothers and partially breastfeeding mothers showed an absolute difference of approximately 0.2 h in mean device-measured total sleep duration (5.4 (SD: 1.5) vs. 5.6 (SD: 1.3) h), although this difference was not significant (*p* = 0.27). Similarly, the proportion of mothers sleeping ≥5 h differed by 4% between the two groups (63.8% vs. 68.0%), but the difference was not significant (*p* = 0.54 tested by Chi2) (Figure 2). In unadjusted analysis, a statistically not significant trend toward shorter device-measured daily sleep duration was seen among exclusive breastfeeding mothers compared to those with partial breastfeeding (unadjusted β = −0.21 h, 95% CI:−0.59, 0.17) (Table 5). After adjusting for socio-demographic, reproductive, and caregiving-related covariates, the association was more attenuated (adjusted β = −0.16 h, 95% CI:−0.56, 0.24). The likelihood of sleeping ≥ 5 h was also similar across feeding groups (adjusted PR = 0.94, 95% CI: 0.65, 1.36).

**Table 5:** Association between device-measured total sleep duration and breastfeeding behaviors among mothers 1 month postpartum (*n* = 197).

*Characteristics*	Device-measured total sleep (hours)				Device-measured sleep ≥5 h (REF:<5 h)			
	Unadjusted β (95% CI)^1^	*p*-val	Adjusted β (95% CI)^2^	*p*-val	Unadjusted Prevalence Ratio (PR) (95% CI)^3^	*p*-val	Adjusted PR (95% CI)^2^	*p*-val
Exclusive breastfeeding	−0.21 (-0.59, 0.17)	0.27	−0.16 (-0.56, 0.24)	0.43	0.94 (0.67, 1.33)	0.72	0.94 (0.65, 1.36)	0.75
Ever pumping breastmilk	−0.32 (-0.85, 0.21)	0.24	−0.07 (-0.63, 0.48)	0.79	0.79 (0.51, 1.24)	0.31	0.86 (0.53, 1.39)	0.55
Daytime breastfeeding frequency	−0.05 (-0.19, 0.10)	0.51	−0.05 (-0.20, 0.09)	0.46	0.99 (0.87, 1.23)	0.88	0.98 (0.85, 1.12)	0.77
Night-time breastfeeding frequency	−0.18 (-0.48, 0.13)	0.26	−0.15 (-0.46, 0.16)	0.34	0.94 (0.71, 1.23)	0.65	0.94 (0.71, 1.25)	0.66
Pumping frequency^4^	0.01 (-0.01, 0.04)	0.27	0.01 (-0.01, 0.04)	0.29	1.01 (0.99, 1.03)	0.53	1.01 (0.99, 1.03)	0.58

^1^Linear regression was used to estimate β coeffcients for the association between breastfeeding behaviors and sleep duration. ^2^Adjusted for women's age, use of maternity leave, delivery mode (normal, c-section), household income, highest education attained, number of children under 5 years old, and ethnicity. ^3^Modified Poisson regression models to estimate prevalence ratios (PR) for the association between breastfeeding behaviors and sleep duration≥5 h. ^4^Tested among mothers who have ever pumped breastmilk (n = 167).

There were no significant differences between other breastfeeding indicators and device-measured total sleep duration, such as daytime breastfeeding frequency (adjusted β = −0.05, 95% CI:−0.20, 0.09), nighttime breastfeeding frequency (adjusted β = −0.15, 95% CI:−0.46, 0.16), ever pumping breastmilk (adjusted β = −0.07; 95% CI:−0.63, 0.48) or pumping frequency (adjusted β = 0.01, 95% CI:−0.01, 0.04).

Findings from sensitivity analyses were generally similar to the primary analysis (Supplemental Table 2); however, a higher pumping frequency showed a marginal association with a greater likelihood of sleeping ≥6 h (adjusted PR = 1.02, 95% CI: 0.99, 1.04). Another sensitivity analysis using mixed-effect models showed consistent results in the association between device-measured total sleep and breastfeeding behaviors with regression models using average sleep duration (Supplemental Table 3).

## Discussion

This study characterized postpartum sleep patterns and explored variation in sleep across breastfeeding contexts among women at 1 month postpartum in Ho Chi Minh, Vietnam. On average, study participants slept 5.5 h per day, which is well below the general recommendation of seven or more hours per night for adults (24).

Exclusive breastfeeding is a proven nutritional intervention that reduces neonatal mortality and prevents morbidity in low- and middle-income countries (25, 28) and is recommended globally (29). In our study, only 47.7% of women maintained exclusive breastfeeding at 1 month postpartum. Most participants breastfed approximately 10 times per day, spending 4.3 h (26.9 min per session x 9.6 times/60 min) on feeding alone, underscoring the time-intensive nature of breastfeeding. Exclusive breastfeeding mothers reported longer total breastfeeding time per day than partially breastfeeding mothers (4.7 vs. 4.0 h).

Maternal sleep duration did not show significant differences between exclusive breastfeeding and partial breastfeeding mothers. The inconsistent differences in sleep duration may reflect broader postpartum caregiving arrangements and feeding contexts rather than breastfeeding practices alone. Postpartum maternal sleep should therefore be interpreted within broader caregiving ecologies rather than as a direct consequence of feeding method alone. Women classified as partially breastfeeding continued to demonstrate a relatively high breastfeeding frequency and duration at 1 month postpartum. The lack of a clear difference between exclusive and partial breastfeeding groups may be explained by the fact that mothers who were partially breastfeeding maintained substantial breastfeeding engagement. Distinctions in specific feeding behaviors between exclusively and partially breastfeeding mothers are expected to become more evident over the postpartum period.

Our findings in urban Viet Nam differ from some research in high-income settings. In a study of 1-month postpartum women in the United States, nocturnal sleep was longer among exclusively breastfeeding women (386 min) than those with some or complete formula feeding (356 min) (4). Similarly, among 2–4 months postpartum women in Shanghai, China, sleep duration was negatively associated with formula-feeding frequency (30). The authors suggested that longer maternal sleep may help maintain breastfeeding through improved milk production. Data from the Australia Time Use Survey of New Mothers (TUSNM) suggest that shorter sleep duration among exclusively breastfeeding mothers resulted from the feeding burden (31). The relationship between maternal sleep duration and feeding patterns is multi-dimensional and may vary across contexts. Nearly all mothers in our study reported co-sleeping with their infant. In many Asian cultural contexts, co-sleeping and room-sharing with infants are common caregiving practices during the postpartum period and may facilitate breastfeeding, whereas independent sleep arrangements are common in Western countries (32). Schindler-Ruwisch et al. reported that sleep arrangements were closely linked with nighttime breastfeeding and affect maternal sleep experiences (33). Hunter et al. suggested that sleep fragmentation may have a greater impact on maternal fatigue and depressive symptoms than total sleep duration alone (34). In our study, frequent daytime napping, repeated nighttime awakenings, and persistent tiredness even after sleeping were prevalent, suggesting substantial sleep disruption and insufficient restorative sleep during the postpartum period.

This study demonstrates the potential of commercial wearable devices for assessing sleep among postpartum women. The study population with high educational attainment in Ho Chi Minh City may have contributed to the easy acceptance of commercial mobile devices, indicating a potential use of the device to promote personal health. The use of Fitbit devices has been extended to diverse populations, including postpartum women, and studies have generally reported acceptable accuracy and high sensitivity (19, 35). Nevertheless, the performance of Fitbit remains subject to variation due to factors such as total sleep duration, analytical approaches, and device-specific settings.

This study has several limitations. First, the cross-sectional nature of the data does not explain the causal relations between sleep duration and breastfeeding behaviors. To address these limitations, a longitudinal study to follow women from early pregnancy to late postpartum is needed. Second, women were recruited from a third-level hospital through convenience sampling, which could have introduced selection bias. Cesarean delivery was common among study participants (53%), with 96.0% of them planned for medical reasons or patient preference (96.0%). This may limit the generalizability of our findings to populations with lower cesarean delivery rates. However, the cesarean section rate observed in our study is consistent with patterns reported in urban tertiary hospitals in Vietnam (36). Third, other characteristics could still influence breastfeeding behaviors. The participants tended to have higher education and income levels compared with the general postpartum population, which may further limit generalizability. Fourth, breastfeeding frequency and duration were based on maternal recall of feeding practices on the previous day and were not validated using feeding diaries or real-time feeding records. While the one-day recall period likely minimized recall bias, measurement error cannot be excluded, Future studies may benefit from incorporating breastfeeding diaries or mobile-based feeding logs to improve the precision of feeding patterns and allow temporal alignment with device-measured sleep data. Fifth, the partial breastfeeding category likely included a heterogeneous range of feeding practices. Detailed information on formula quantity, timing of formula feeds, and nighttime formula use was not collected. Consequently, mothers classified as partially breastfeeding may have differed substantially in their actual feeding patterns, which could have attenuated associations between breastfeeding practices and sleep outcomes. Our data about sleeping arrangements was incomplete; nighttime delegation or caregiving support from other family members was not included. Sixth, the use of wearable devices and participants' access to their own sleep data may have influenced breastfeeding and sleep behaviors. Since participants could access their own sleep data, their awareness of their sleep quality might have influenced their efforts to improve it. However, there is little evidence to suggest that such reactivity differed by breastfeeding practice (i.e., exclusive versus partial breastfeeding), and therefore, it is unlikely to have introduced differential bias into the association between breastfeeding and sleep quality. Finally, this study focused only on total sleep duration as the primary parameter. Other important aspects of sleep health, such as sleep efficiency and subjective sleep quality, were not assessed. It is possible that even with shorter total sleep duration, sleep efficiency or overall perceived quality may remain similar across breastfeeding practices.

### Further study

Further research will explore associations between various sleep components, such as deep, light, and REM sleep, and breastfeeding practices. In the present study, breastfeeding was self-reported in average hours, whereas device-measured sleep was recorded to the minute. Sleep data were collected only at 1 month postpartum. To better understand the relationship between early postpartum sleep patterns and long-term health outcomes, including postpartum depression, nutritional status, noncommunicable disease risk, and physical activity, longitudinal follow-up studies are warranted. Building on these findings, future research will investigate how maternal sleep patterns impact children's sleep and physical activity. The results will provide evidence to strengthen maternal health and inform breastfeeding. Further study can consider postpartum women in rural areas of LMICs, where findings may differ from those observed among urban mothers.

## Conclusion

This study explored postpartum sleep duration and behaviors and examined the association between breastfeeding patterns and sleep duration among 1-month postpartum women in Ho Chi Minh City, Vietnam. Mothers in this study experienced postpartum sleep restriction regardless of breastfeeding practice. No statistically significant associations in maternal sleep duration were observed between exclusive and partial breastfeeding groups, and breastfeeding frequency and pumping practices were not significantly associated with maternal sleep duration. Because sleep and feeding behaviors occur within complex postpartum contexts that were only partially captured in this study, further longitudinal research with more detailed assessments of caregiving arrangements and feeding practices is warranted.

## Data Availability

The data that support the findings of this study are not publicly available due to ethical restrictions and participant confidentiality. Data may be available from the corresponding author upon reasonable request and with approval from the Ethics Committee of the University of Medicine and Pharmacy at Ho Chi Minh City. Requests to access the datasets should be directed to ykang12@snu.ac.kr.
