# Supplementary material for: Device-measured postpartum sleep and breastfeeding contexts among women in urban Vietnam
Source: Front Public Health. 2026 Aug 25;14:1887659. doi: 10.3389/fpubh.2026.1887659 (PMC13550006; doi:10.3389/fpubh.2026.1887659)
Supplement: Supplementary file 1 [file Table_1.docx]

**Supplemental Table 1. Caregiving environment among exclusive and partial breastfeeding women (n=197)**

| Characteristics | All (n=197) | Exclusive breastfeeding  (n=94) | Partial breastfeeding  (n=103) | p-val^1^ |
| --- | --- | --- | --- | --- |
| Preparing family meals (cooking, setting a table, washing dishes) | | | | |
| <30 minutes | 73 (37.1) | 40 (42.5) | 33 (32.0) | 0.32 |
| 30-<1 hour | 45 (22.8) | 21 (22.3) | 24 (23.3) |  |
| 1-<2 hours | 1 (0.5) | 1 (1.1) | 0 (0.0) |  |
| 2-<3 hours | 1 (0.5) | 0 (0.0) | 1 (1.0) |  |
| None | 77 (39.1) | 32 (34.0) | 45 (43.7) |  |
| Cleaning (make beds, laundry, iron, put things away, vacuum, mop, sweep, wash windows) | | | | |
| <30 minutes | 86 (43.6) | 40 (42.5) | 46 (44.7) | 0.76 |
| 30-<1 hour | 36 (18.3) | 17 (18.1) | 19 (18.4) |  |
| 1-<2 hours | 0 (0.0) | 0 (0.0) | 0 (0.0) |  |
| 2-<3 hours | 1 (0.5) | 1 (1.1) | 0 (0.0) |  |
| None | 74 (37.6) | 36 (38.3) | 38 (36.9) |  |
| Dressing, bathing, and feeding children | | | | |
| <30 minutes | 5 (2.5) | 2 (2.1) | 3 (2.9) | 0.15 |
| 30-<1 hour | 23 (11.7) | 8 (8.5) | 15 (14.6) |  |
| 1-<2 hours | 88 (44.7) | 36 (38.3) | 52 (50.5) |  |
| 2-<3 hours | 74 (37.6) | 43 (45.7) | 31 (30.1) |  |
| >3 hours | 6 (3.0) | 4 (4.3) | 2 (2.0) |  |
| None | 0 (0.5) | 1 (1.1) | 0 (0.0) |  |
| Playing with children | | | | |
| <30 minutes | (0.0) | 0 (0.0) | 0 (0.0) | 0.13 |
| 30-<1 hour | 5 (2.5) | 1 (1.1) | 4 (3.9) |  |
| 1-<2 hours | 60 (30.5) | 23 (24.5) | 37 (35.9) |  |
| 2-<3 hours | 119 (60.4)) | 61 (64.9) | 58 (56.3) |  |
| >3 hours | 13 (6.6) | 9 (9.6) | 4 (4.0) |  |
| None | 0 (0.0) | 0 (0.0) | 0 (0.0) |  |
| Carrying children | | | | |
| <30 minutes | 1 (1.1) | 1 (1.1) | 2 (1.9) | 0.10 |
| 30-<1 hour | 6 (6.4) | 6 (6.4) | 18 (17.5) |  |
| 1-<2 hours | 140 (71.1) | 73 (77.7) | 67 (65.0) |  |
| 2-<3 hours | 11 (11.7) | 11 (11.7) | 15 (14.6) |  |
| >3 hours | 3 (3.2) | 3 (3.2) | 1 (1.0) |  |
| None | 6 (6.4) | 6 (6.4) | 18 (17.5) |  |
| Taking care of older adults and other members | | | | |
| <30 minutes | 5 (2.5) | 2 (2.1) | 3 (2.9) | 0.63 |
| 30-<1 hour | 1 (0.5) | 0 (0.0) | 1 (1.0) |  |
| 1-<2 hours | 1 (0.5) | 0 (0.0) | 1 (1.0) |  |
| 2-<3 hours | 1 (0.5) | 0 (0.0) | 1 (1.0) |  |
| None | 133 (67.5) | 67 (71.3) | 66 (64.1) |  |
| Not applicable | 56 (28.4) | 25 (26.6) | 31 (30.1) |  |
| Sitting or resting in a day | | | | |
| <30 minutes | 12 (6.1) | 4 (4.3) | 8 (7.8) | 0.13 |
| 30-<1 hour | 95 (48.2) | 39 (41.5) | 56 (54.4) |  |
| 1-<2 hours | 79 (40.1) | 44 (46.8) | 35 (34.0) |  |
| 2-<3 hours | 9 (4.6) | 5 (5.3) | 4 (3.9) |  |
| >3 hr | 2 (1.0) | 2 (2.1) | 0 (0.0) |  |
| None | 0 (0.0) | 0 (0.0) | 0 (0.0) |  |
| Shopping for foods, clothes, or other items | | | | |
| <30 minutes | 134 (68.0) | 65 (69.2) | 69 (67.0) | 0.23 |
| 30-<1 hour | 20 (10.1) | 13 (13.8) | 7 (6.8) |  |
| 1-<2 hours | 3 (1.5) | 1 (1.1) | 2 (1.9) |  |
| None | 40 (20.3) | 15 (16.0) | 25 (24.3) |  |

**Supplemental Table 2. Sensitivity analysis: Association between device-measured 6 or 7 hours sleep duration and breastfeeding behaviors among mothers one month postpartum (n=197)**

| ***Characteristics*** | Device-measured sleep ≥6 hours (REF: <6 hours) | | | | Device-measured sleep ≥7 hours (REF: <7 hours) | | | |
| --- | --- | --- | --- | --- | --- | --- | --- | --- |
|  | Unadjusted prevalence ratio (PR) (95%CI)^1^ | p-val | Adjusted PR (95%CI)^2^ | p-val | Unadjusted PR (95%CI)^1^ | p-val | Adjusted PR (95%CI)^1^ | p-val |
| Exclusive breastfeeding | 0.90 (0.57, 1.43) | 0.67 | 0.94 (0.57, 1.54) | 0.80 | 0.63 (0.26, 1.49) | 0.29 | 0.62 (0.24, 1.57) | 0.32 |
| Ever pumping breastmilk | 1.01 (0.53, 1.92) | 0.97 | 1.20 (0.60, 2.42) | 0.61 | 1.80 (0.42, 7.69) | 0.43 | 2.72 (0.56, 13.2) | 0.21 |
| Daytime breastfeeding frequency | 0.92 (0.78, 1.09) | 0.36 | 0.92 (0.77, 1.09) | 0.33 | 0.97 (0.71, 1.34) | 0.87 | 0.99 (0.70, 1.39) | 0.95 |
| Night-time breastfeeding frequency | 0.91 (0.63, 1.30) | 0.60 | 0.94 (0.64, 1.38) | 0.76 | 0.96 (0.49, 1.88) | 0.91 | 1.05 (0.50, 2.21) | 0.90 |
| Pumping frequency^3^ | 1.05 (1.00, 1.04) | 0.05 | 1.02 (0.99, 1.04) | 0.06 | 1.01 (0.97, 1.06) | 0.51 | 1.01 (0.97, 1.06) | 0.50 |

^1^ Modified Poisson regression models to estimate prevalence ratios (PR) for the association between breastfeeding behaviors and sleep duration≥5 hours.

^2^ Adjusted for women’s age, use of maternity leave, delivery mode (normal, c-section), household income, highest education attained, number of children under five years old, and ethnicity.

^3^ Tested among mothers who have ever pumped breastmilk (n=167).

**Supplemental Table 3. Sensitivity analysis: Association between device-measured sleep duration and breastfeeding behaviors among mothers one month postpartum (n=197) by mixed-effect analysis**

| ***Characteristics*** | Device-measured total sleep (hours) | | | |
| --- | --- | --- | --- | --- |
|  | Unadjusted β (95%CI)^1^ | p-val | Adjusted β (95%CI)^2^ | p-val |
| Exclusive breastfeeding | -0.16 (-0.53, 0.20) | 0.37 | -0.10 (-0.48, 0.27) | 0.59 |
| Ever pumping breastmilk | -0.34 (-0.84, 0.15) | 0.17 | -0.09 (-0.61, 0.42) | 0.72 |
| Daytime breastfeeding frequency | -0.04 (-0.18, 0.09) | 0.53 | -0.04 (-0.18, 0.09) | 0.53 |
| Night-time breastfeeding frequency | -0.10 (-0.39, 0.19) | 0.52 | -0.09 (-0.38, 0.20) | 0.54 |
| Pumping frequency^2^ | 0.01 (-0.01, 0.03) | 0.35 | 0.01 (-0.01, 0.03) | 0.35 |

^1^β-coefficients were estimated using linear mixed-effect models with breastfeeding behaviors as fixed effects and participant as a random intercept.

^2^Adjusted models accounted for women’s age, use of maternity leave, delivery mode (normal or cesarean section), household income, highest education attained, number of children under five years old, ethnicity, and measurement day
